# Neutrophil Infiltration of the Colon Is Independent of the FPR1 yet FPR1 Deficient Mice Show Differential Susceptibilities to Acute Versus Chronic Induced Colitis

**DOI:** 10.1007/s10620-012-2082-y

**Published:** 2012-03-02

**Authors:** Shukkur M. Farooq, Andrew W. Stadnyk

**Affiliations:** 1 Department of Pediatrics, Dalhousie University, Halifax, NS Canada; 2 Department of Microbiology and Immunology, Dalhousie University, Halifax, NS Canada; 3 Present Address: Department of Pharmacy Practice, Eugene Applebaum College of Pharmacy and Health Sciences, Wayne State University, 259 Mack Avenue, Detroit, MI 48201 USA; 4 Mucosal Immunology Research, IWK Health Center, 5850 University Avenue, Halifax, NS B3K 6R8 Canada

**Keywords:** Formyl peptide receptor, Neutrophil, Colitis, Dextran sodium sulphate, Inflammation

## Abstract

**Background:**

The receptor for formylated peptides, formyl peptide receptor 1 (FPR1), potently activates and serves as a chemoattractant receptor for neutrophils.

**Aim:**

Given the abundance of neutrophils in the inflamed colon, our aim was to determine if the FPR1 mediates colonic neutrophil migration, using the dextran sodium sulfate (DDS)-induced model of colitis.

**Methods:**

Formyl peptide receptor 1 gene-deficient mice were administered DDS in drinking water for a single 5-day period (acute) or in two 5-day periods separated by 16 days (chronic). At the end of the treatment their colons were excised, measured, and prepared for histological evaluation.

**Results:**

FPR1^−/−^ mice experienced less severe acute colonic pathology than C57BL/6 (wildtype) mice. The opposite was observed following the second colitis cycle, with FPR1^−/−^ mice developing worse pathology than wildtype mice. Both strains had similar numbers of infiltrating neutrophils in ulcerated areas of the colon after a single DSS cycle, but FPR1^−/−^ mice had significantly more neutrophils in the ulcerated mucosa after two cycles. There was no difference in the capacity of neutrophils from each strain to migrate to chemoattractants. Since the FPR1^−/−^ mice had larger ulcers compared to the wildtype mice, we propose that the FPR1^−/−^ mice failed to recover at the same rate as wildtype mice. This apparent difference in restitution could not be attributed to observable differences in annexin A1.

**Conclusions:**

We conclude that neutrophil migration into the inflamed mouse colon does not depend on FPR1 but that FPR1 contributes in other pathological mechanisms that are harmful during acute inflammation but protective during chronic inflammation.

## Introduction

Bacterial protein translation is initiated with *N*-formyl methionine, which in many proteins is cleaved post-translationally, resulting in the release of low-molecular-weight formylated peptides, including those by bacteria from the intestines [1]. Mammalian cells detect formylated peptides with a family of seven membrane-spanning G-protein coupled receptors, the principal one being formyl peptide receptor 1 (FPR1). This innate immune receptor was first described on neutrophils that were found to become activated by binding the prototypic formyl peptide, *N*-formyl-methionyl-phenylalanine (fMLF) [2–4]. Given the abundance of bacteria in the colon, there is good reason to believe that formyl peptides play a role in recruiting neutrophils into intestinal inflammatory diseases.

While commensal organisms generate formyl peptides, whether these contribute to gastrointestinal inflammation has not been directly shown. Instead, researchers have applied fMLF to the rodent intestinal lumen, with mixed outcomes. In one example, fMLF applied in a small intestinal perfusate resulted in increased microvascular permeability, yet had no effect on neutrophil numbers [5, 6]. In a second example, the fMLF resulted in marked neutrophil infiltration in the perfused segment [7]. Other researchers have added fMLF to rats experiencing experimentally induced colitis, thereby exacerbating the colitis [5, 8]. Exacerbated experimental colitis may indicate that a breach of the epithelium must first occur to permit effective diffusion of the peptide into the mucosa. However, the finding of increased neutrophil numbers when fMLF is applied to the healthy rodent colon is inconsistent, and in none of these studies was it assessed whether endogenous levels of formyl peptides act to recruit neutrophils using FPR1.

Another approach to address the question of which chemoattractants may be active is to use mice deficient for chemoattractant receptors. We and others have previously used mice deficient for the chemokine receptor CXCR2 to gain insights into the role this receptor plays in neutrophil recruitment into the colon [9, 10]. The CXCR2 gene knockout strain had reduced numbers—but not the ablation—of neutrophils in the mucosa of the dextran sodium sulfate (DSS) inflamed colon, leading to the conclusion that this family of chemokine ligands does not work alone. In light of evidence that exogenous fMLF enhances colonic neutrophil infiltration during induced colitis and that the neutrophils of patients with inflammatory bowel disease possess increased numbers of FPR1 [11], we thought it interesting to examine FPR1 gene-deficient mice with DSS-induced colitis. We report here that the gene deficiency has little impact on neutrophil numbers infiltrating the acutely inflamed colon, but it does result in heightened numbers of cells infiltrating the chronically inflamed colon, which is associated with more severe disease.

## Materials and Methods

### Animals

Formyl peptide receptor 1 gene-deficient mice on the C57BL/6 genetic background were kindly provided by Dr. P. Murphy (NIAID, National Institutes of Health, Bethesda, MD) [12]. The strain was bred in our facility and on two occasions over 24 months were bred with C57BL/6 mice (Charles River Laboratories, Saint-Constant, QC, Canada), then back to homozygosity for the knockout gene. C57BL/6 mice served as wildtype controls. All animal experiments were approved by the Dalhousie University Committee on Laboratory Animals, which adjudicates the guidelines of the Canadian Council on Animal Care.

### DSS Colitis and Clinical Scoring

To induce acute colitis, we added 4% DSS (36,000–50,000 MW; MP Biomedicals, Solon, OH) to the animals’ water for 5 days, followed by facility water until the ninth day when they were euthanized. For the chronic studies, mice received 4% DSS for 5 days, followed by facility water until day 22, after which they were once again given 3% DSS for 5 days; they were euthanized on day 34. Mice were weighed throughout the experiment, and the consistency of their stool and whether blood was present in their stool were recorded (Hemoccult kit; Beckman-Coulter, Mississauga, ON, Canada).

### Pathology Scores and Explant Cultures

At the end of the experiments, mice were euthanized by CO_2_ asphyxiation and their colons removed and the length measured. The colons were opened longitudinally along the mesenteric axis, bisected longitudinally, and one half was prepared in a Swiss roll and fixed in buffered formalin. The fixed tissue was prepared in paraffin and 3-µm-thick sections were cut and stained using hematoxylin and eosin. The stained sections were assessed for pathology by two blinded researchers using a scale that takes into account the presence (or not) of submucosal edema, the extent of mucosal ulceration, crypt loss, and infiltration (for a more detailed description of the scale, see [10]). The second half of the dissected colon was weighed, then washed repeatedly in RPMI media with antibiotics (50 U/ml penicillin and 50 μg/ml streptomycin; Life Technologies, Burlington, ON, Canada) and incubated in media containing antibiotics and 10% fetal calf serum. The supernatants were recovered after 24 h and stored frozen until assayed for macrophage inflammatory protein 2 (MIP-2) using an enzyme-linked immunosorbent asssay (ELISA; PeproTech Canada, Dollard des Ormeaux, QC, Canada). The results were expressed as: [total MIP-2 divided by the weight of the colon (mg)] × 100.

### Immunohistochemistry

Mouse colon sections were de-paraffinized, and antigen retrieval was performed in sodium citrate buffer. Endogenous peroxidases were first blocked using 3% H_2_O_2_, followed by blocking the sections using 5% goat serum–phosphate buffered saline (PBS)/Tween. The sections were incubated with rat anti-mouse Ly6G (BD Biosciences, San Jose, CA) or anti-annexin A1 (Santa Cruz Biotechnology, Santa Cruz, CA) overnight at 4°C. The sections were then washed and incubated with biotin conjugated goat anti-rat antiserum (Santa Cruz Biotechnology) for 1 h at room temperature, followed by further washes. Avidin–biotin complex (Vector Laboratories, Burlingame, CA) was added for 30 min at room temperature, following which the sections were incubated with 3,3-diaminobenzidine (Amersham Biosciences, Piscataway, NJ) for 10 min at room temperature, washed, counterstained with Mayer’s hematoxylin, and processed for mounting.

### Ex vivo Neutrophils Migration Assay

Neutrophils recovered from the fibia and tibia of mice were used in Transwell migration assays essentially as described elsewhere for human cells [13]. Briefly, bone marrow were enriched using Percoll gradients and radiolabeled with Na_2_^51^CrO_4_ (Amersham, Oakville, ON, Canada); 10^5^ cells were then applied to the upper Transwell filter chamber (pore size of filter 4 μm), with the chemoattractant in the bottom chamber. After a 90-min incubation, the filter and upper chamber were removed and the filter bottom washed to remove any loosely adherent cells. These cells were pooled with cells that had migrated into the bottom chamber and all cells were lysed with 1% Triton X-100 and the amount of radioactivity counted in an automatic gamma counter (Wallac, Turku, Finland). The migrated fraction of neutrophils was expressed as a percentage of the total input number of neutrophils.

### Western Blotting

The entire colon was cut open longitudinally and washed twice with cold PBS. A 500-μl aliquot of lysis (M2) buffer was added onto the mucosal side for 5 min, following which the mucosa was scraped using a scalpel. The recovered solution was transferred to a cold microfuge tube and vortex shaken gently for 1 min. Protein concentrations were determined (BioRad, Hercules, CA) before separation by sodium dodecyl sulfate-polyacrylamide gel electrophoresis (SDS-PAGE). Both cell lysates and protein standards (Bio-Rad) were mixed with SDS sample buffer and resolved by 7.5–10% SDS-PAGE. Following electrophoresis, the proteins were electro-transferred onto nitrocellulose membranes, and the filters were incubated with 5% non-fat milk in TRIS buffered saline for 1 h and then processed for immunolabeling. Membranes were probed with a rabbit anti-mouse Annexin A1 antiserum (Santa Cruz) overnight at 4°C. β-actin probing was used as a loading control. Horseradish peroxidase-labeled goat anti-rabbit antiserum was used as the secondary reagent at room temperature for 1 h. The immunoreactivity was visualized with chemiluminescence (Amersham Pharmacia Biotech, Buckinghamshire, UK) and subsequent exposure to X-ray film.

### Statistical Analyses

Data were expressed as the mean ± standard error of the mean (SEM). Differences in histopathologic scoring were evaluated by the Mann–Whitney *U* test, while all other measures were analyzed using the two-tailed Student’s *t* test (SPSS ver. 14.0; SPSS, Chicago, IL). Statistical significance was set at a *p* value of  ≤0.05.

## Results

### FPR1^−/−^ Mice Show Different Patterns of Weight Loss Following One Versus Two DSS Cycles

**Fig. 1:**
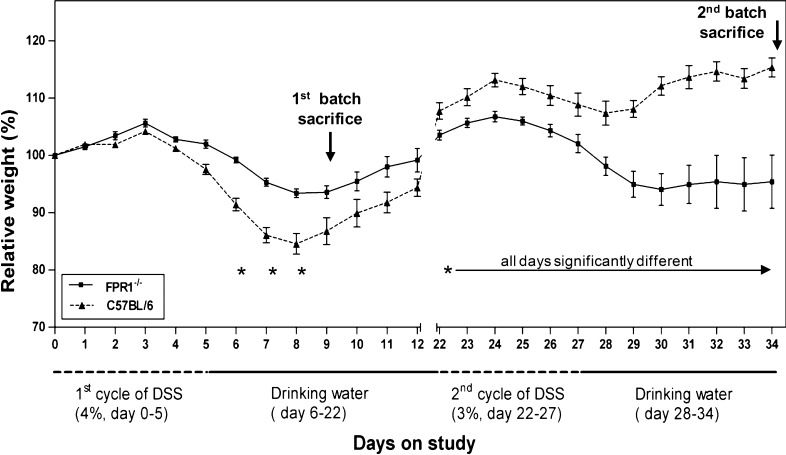
Pattern of weight changes in C57BL/6 and formyl peptide receptor 1-deficient (*FPR1* ^*−/−*^) mice receiving one (acute) or two (chronic) cycles of dextran sodium sulfate (*DSS*) in their drinking water. Data are presented as the mean and standard error of the mean (SEM) of their percentage weight relative to the each respective animal’s starting weight. *n* = 8 mice of each strain, conducted in two experiments.* Asterisk* indicates a statistically significant difference between strains

During the first cycle of DSS treatment a decrease in body weight was observed in both strains of mice (Fig. 1). The average maximum weight loss among C57BL/6 and FPR1^−/−^ mice was 14 and 5%, respectively. The C57BL/6 mice developed diarrhea and gross blood in their stool, while FPR1^−/−^ mice had soft stools with occult blood but no diarrhea. The difference in weight loss between the strains was significant on days 6, 7, and 8. Three days after the DSS treatment was discontinued, both strains began to gain weight. By day 22, the C57BL/6 mice had gained more weight than the FPR1^−/−^ mice (Fig. 1). During the second DSS cycle the FPR1^−/−^ mice experienced a greater weight loss than the C57BL/6 mice as well as more severe bloody diarrhea, and they failed to gain weight after the DSS was discontinued. The weight differences were statistically significant between and including days 22–34 (Fig. 1).

### Histopathology Following One DSS Cycle: FPR1^−/−^ Mice Show Less Inflammation than C57BL/6 mice

**Fig. 2:**
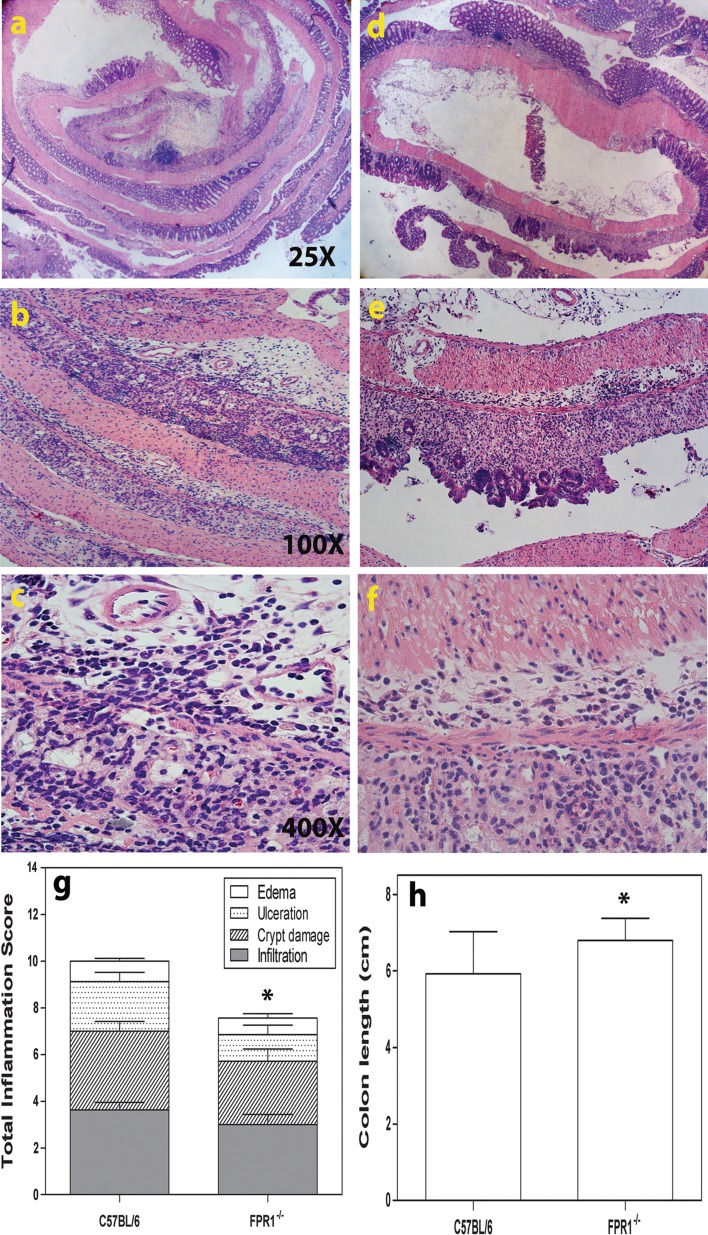
Representative images showing colon histopathology of acutely inflamed mice sacrificed on study day 9. **a**, **b**, **c** Increasing magnifications of the same section from a C57BL/6 mouse, **d**, **e**, **f** increasing magnifications of the same section from a FPR1^−/−^ mouse. **g** The average for each element in the histopathological (inflammation) score for the two strains. The difference between the sums of each mouse strain’s pathology scores in **g** is statistically different by Mann–Whitney *U* test. **h** Colon lengths. Differences in colon lengths between the two strains were statistically significant (*p* < 0.05) according to Student’s *t* test (*asterisk*). In **g** and **h**, *n* = 8 mice of each strain. *Bars* Mean and SEM

Histological examination of the colons of C57BL/6 mice administered 4% DSS revealed submucosal edema with dilated blood vessels, ulceration, extensive mononuclear cell and neutrophil infiltration, and the loss of crypts at multiple sites of the mid-colon (Fig. 2a–c). In comparison, histological examination of the FPR1^−/−^ colon showed stretches with submucosal edema but with less ulceration, infiltration, and crypt damage than in C57BL/6 mice (Fig. 2d–f). The histopathology scores were statistically significantly different between the strains (Fig. 2g) as were their colon lengths (Fig. 2h).

### Following Two DSS Cycles, FPR1^−/−^ Mice Show More Inflammation than C57BL/6

**Fig. 3:**
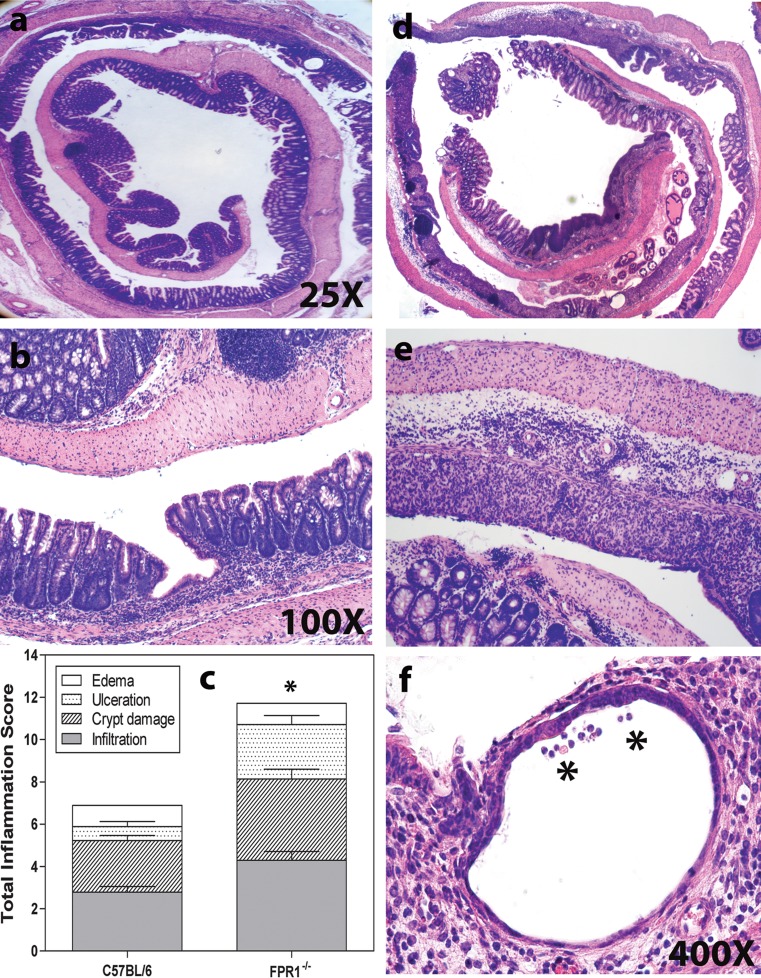
Representative images showing the colon histopathology of chronically inflamed mice sacrificed on study day 34. **a**, **b** C57BL/6 mice, **d**, **e**, **f** FPR1^−/−^ mice. **f** *Asterisk* denotes leukocytes in a crypt. **c** Histopathological scoring for the two strains. *Bars*: Mean and SEM of each element of the pathology scale, with *n* = 8. The difference between the sums of each mouse strain’s pathology scores in **c** is statistically significant at *p* < 0.05 according to the Mann–Whitney *U* test

C57BL/6 mice showed less pathology at the end of the experiments following the second DSS cycle than they did after the first cycle, including smaller scattered ulcers (Fig. 3a, b). The large weight loss among FPR1^−/−^ mice indicated that they might have worse colon histopathology, and indeed these mice showed considerable submucosal edema and mucosal ulceration with mononuclear and neutrophil infiltration, including crypt abscesses, in multiple sites affecting upwards of 75% of this stretch of colon (Fig. 3d–f). The histopathology scores were significantly different (Fig. 3c), as were the colon lengths (not shown).

### Inflamed FPR1^−/−^ Mice Exhibit Neutrophil Infiltration of the Colon

**Fig. 4:**
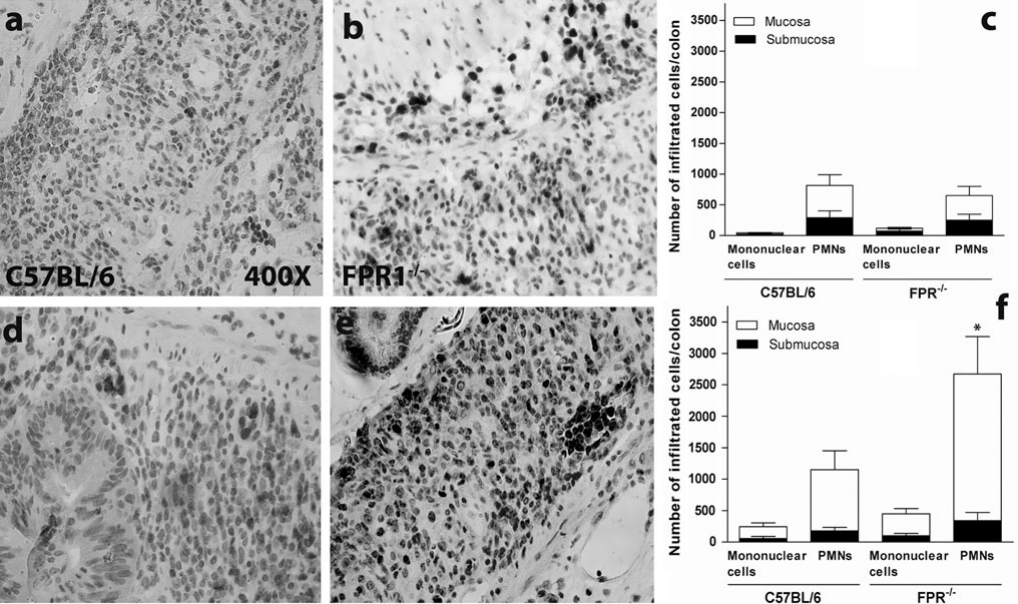
Ly6G immunohistochemical staining identifies neutrophils (polymorphonuclear leukocytes,* PMNs*) in the inflamed colon sections. Representative images showing colon histopathology on day 9. **a**, **d** C57BL/6 mice, **b**, **e** FPR1^−/−^ mice. **c** The average Ly6G^+^ cell count from mice killed on day 9. Ly6G^+^ cells with polymorphic nuclei (neutrophils) and mononuclear cells were counted in the mucosa versus submucosa throughout the entire colon on a single section of each mouse of both strains. **f** The average Ly6G^+^ cell count from mice killed on day 34. The difference between the number of mucosal neutrophils in the chronically inflamed C57BL/6 versus FPR1^−/−^ mice is statistically significant at *p* < 0.05 (*asterisk*)

Neutrophils are a hallmark infiltrating leukocyte in the DSS-induced inflamed colon. As we are interested in knowing whether the FPR1 plays a role in the inflammation process, we stained sections for Ly6G [14]. In our experience, some of the Ly6G^+^ cells in the inflamed mouse colon are mononuclear, allowing these cells to be distinguished from PMN under high magnification. The healthy mouse colon had only a few scattered Ly6G^+^-positive cells (not shown), but mucosal and submucosal infiltration occurred in both strains following DSS treatment, with no significant difference between strains (Fig. 4a–c). After the second DSS cycle, both strains again showed high numbers of Ly6G^+^ PMN, but the FPR1^−/−^ mice had significantly greater numbers than the C567BL/6 mice (Fig. 4d–f). Both strains had similar numbers of infiltrating Ly6G^+^ mononuclear cells (Fig. 4c, f).

### Strain Differences in Neutrophil Infiltration Are Not Due to Intrinsic Differences in the Response to Chemoattractants

**Fig. 5:**
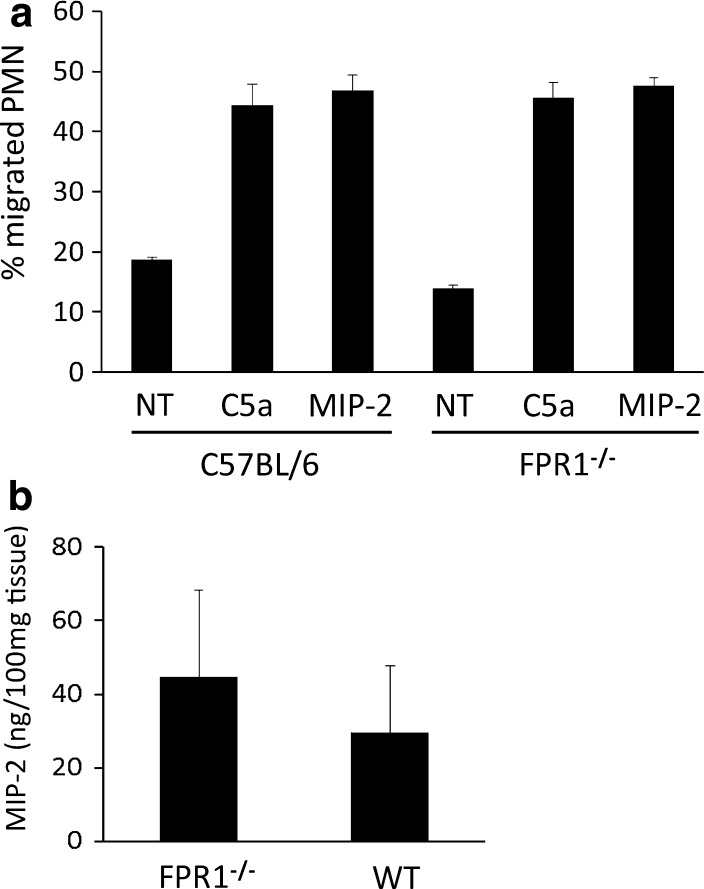
Neutrophils from inflamed FPR1^−/−^ mice respond to chemoattractants in a similar manner as neutrophils from inflamed C57BL/6 mice. Neutrophils were enriched to 95% from the bone marrow of both strains 22 days after starting a DSS cycle (acutely inflamed, as in Fig. 1) **a** Neutrophils were tested for migration to 10^−8^ M human recombinant C5a (protein fragment of complement component C5) and 10^−8^ M recombinant macrophage inflammatory protein 2 (*MIP-2*). *Bars*: Mean ± SD, *n* = 4 mice, one of two experiments (conducted using mice sacrificed on day 21). **b** MIP-2 levels in 24 h ex vivo colon culture supernatants from the same mice as in **a** (e.g., following one cycle of DSS).* WT* Wildtype (C57BL/6)

We were interested in determining whether the increase in neutrophil infiltration may be due to an intrinsic difference (increase) in the chemotactic response by neutrophils from FPR1^−/−^ mice, particularly after the first cycle of DSS. Bone marrow cells were isolated from mice 22 days after the start of the first DSS cycle, e.g., on the same day that the second DSS cycle was started (Fig. 1). The neutrophils were enriched on Percoll density gradients to 95% purity, following which the cells were radiolabeled and applied to the top of a Transwell filter chamber. Migration of the cells through the filter into the bottom chamber was then induced by placing a chemoattractant in the bottom chamber. Figure 5a shows that both C5a (protein fragment from complement component C5) and MIP-2 recruited neutrophils from both strains, without any difference in potency, leading us to conclude no obvious alteration had occurred in the neutrophils from inflamed FPR1^−/−^ mice that might explain their heightened infiltration during the second DSS cycle. From the same inflamed mice we recovered the colon and, following extensive washes in antibiotic-containing media, cultured the tissue overnight in order to recover the secreted chemoattractants. The MIP-2 concentrations in these supernatants are shown in Fig. 5b. MIP-2 concentrations of healthy mouse colons were <3.5 ng/ml, essentially off the scale of units in Fig. 5b. In contrast, MIP-2 levels were significantly elevated in both strains when inflamed compared to their healthy controls. However, despite the appearance of higher MIP-2 concentrations in FPR1^−/−^ mice, there was no statistically significant difference between the inflamed strains.

### Inflamed FPR1^−/−^ Mice Show Severe Ulcers yet Wound Healing Proceeds

**Fig. 6:**
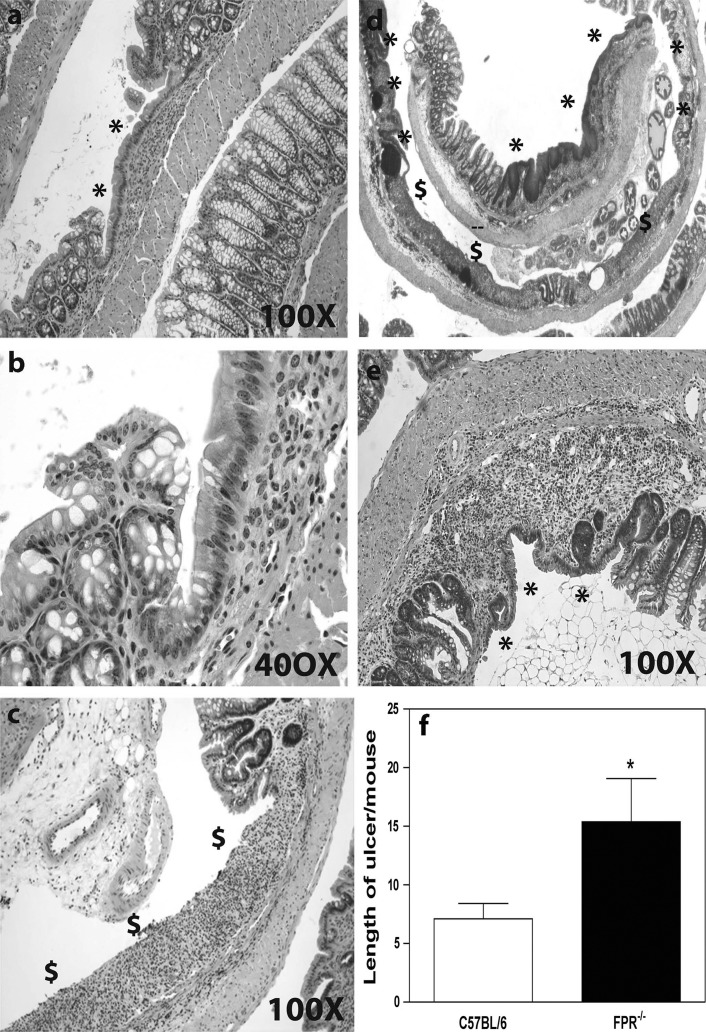
Colon sections from chronically inflamed mice showing the extent of restitution or re-epithelialization of ulcerated regions. **a** Epithelium has entirely crowned the damaged mucosa in this example from a C57BL/6 mouse, identified by the* asterisk*. **b** A higher magnification image of the ulcer margin taken from the image in **a**. **c** An ulcer remains without epithelium on a long stretch of damaged mucosa in the FPR1^−/−^ mouse. **d** Low-power micrograph of a second FPR1^−/−^ mouse colon showing mixed extent of restitution;* asterisk* denotes re-epithelialized ulcers,* dollar sign* non-epithelialized ulcers. **e** A higher magnification example of a completely re-epithelialized region of damaged crypts in an FPR1^−/−^ mouse. **f** Average ulcer length in the two strains reveals that ulcers are longer in the FPR1^−/−^ mice, with the difference statistically significantly different at *p* < 0.05 (*asterisk*)

In a further characterization of the differences between C57BL/6 and FPR1^−/−^ colons, we observed that following the second DSS treatment stretches of the mucosa with crypt loss in the C57BL/6 mice were completely or near completely re-epithelialized (Fig. 6a, b). Contrasting this, and with the exception of small ulcers, most ulcers in FPR1^−/−^ mice were incompletely re-epithelialized (Fig. 6c–e). Measurements of the ulcers demonstrated that on average the ulcers were longer (larger) in FPR1^−/−^ mice than in C57BL/6 mice (Fig. 6f).

### Mucosal Annexin A1 Levels Are Similar Between the Two Strains of DSS-Inflamed Mice

**Fig. 7:**
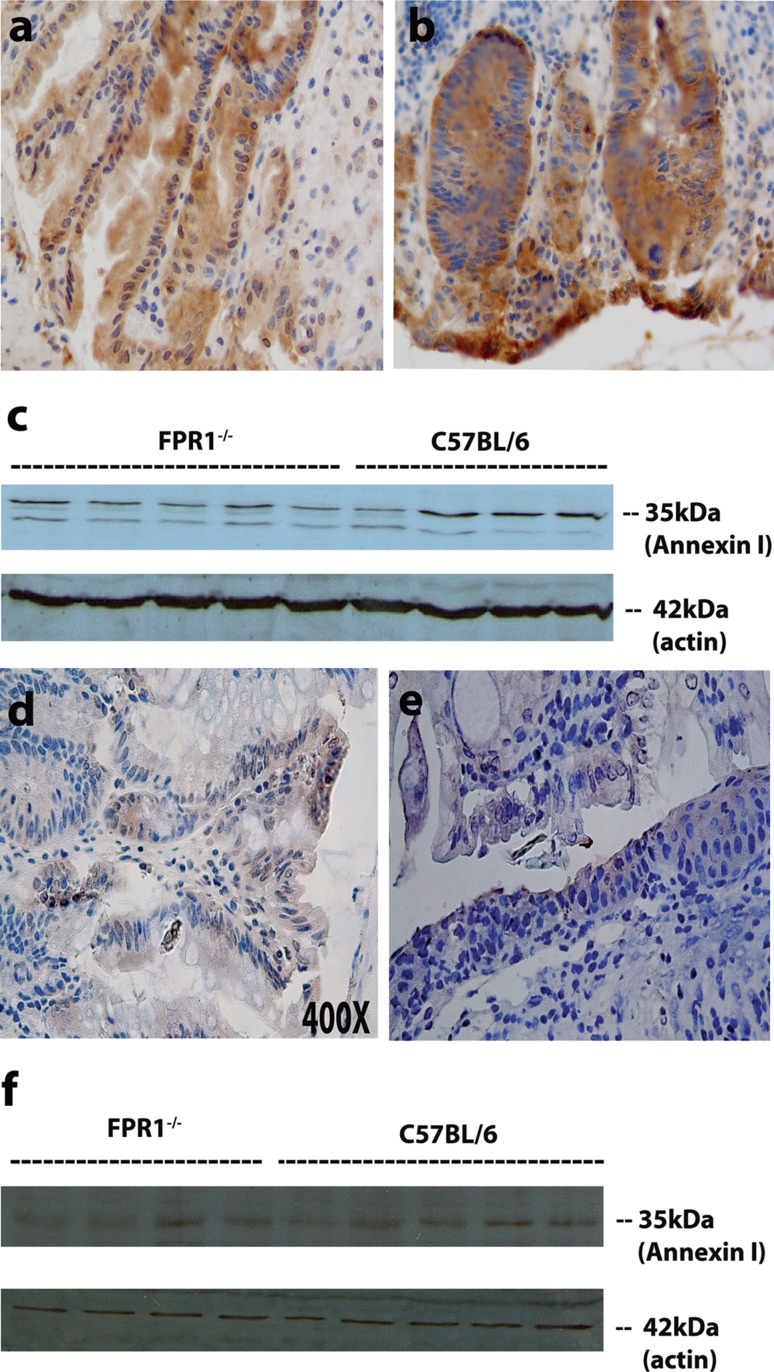
Immunohistochemical detection of annexin A1 in inflamed mice. **a** Representative acutely inflamed C57BL/6 mouse, **b** representative acutely inflamed FPR1^−/−^ mouse, **c** Western blot for colonic annexin A1 and a protein loading control (β-actin) for acutely inflamed mice, **d** immunohistochemical detection of annexin A1 in a representative chronically inflamed C57BL/6 mouse, **e** representative chronically inflamed FPR1^−/−^ mouse, **f** Western blot for colonic annexin A1 protein for multiple chronically inflamed mice and the actin loading control

Formyl peptides acting through FPR1 on neutrophils promote inflammation by recruiting cells and causing degranulation, but FPR1 can be anti-inflammatory when annexin A1 binds and inhibits neutrophil extravasation [15]. Annexin A1 can also directly regulate the recovery from intestinal mucosal injury [16] and repair of the gastric mucosa [17] by acting on epithelial cells, which have recently been found to express formyl peptide receptors. In this context, autocrine annexin A1 acts through the FPRL1 to promote epithelial cell migration. Our results showing the presence of copious numbers of neutrophils and larger ulcers in FPR1^−/−^ mice may be related to a reduction in these anti-inflammatory activities of annexin A1, leading us to examine annexin A1 levels in the mice. Figure 7 shows that mucosal annexin A1 levels following a single DSS cycle were similar in the two strains. Noteworthy, annexin A1 observed by immunohistochemistry was reduced in both strains following the second DSS cycle (Fig. 7d–f) compared to the first cycle (Fig. 7a–c). Hence, a strain-specific lack of annexin A1 expression is unlikely to explain the exacerbation of ulcer formation or reduced re-epithelialization in FPR1^−/−^mice.

## Discussion

The family of formyl peptide receptors comprises three members, i.e., FPR1, FPRL1, and FPRL2. Of these, the FPR1 of humans is considered to be the principal receptor for fMLF. On neutrophils, FPR1 represents a potent receptor, leading to cell activation and chemokinesis. In actual fact, the migration of human neutrophils to fMLF desensitizes other G-protein-coupled receptors involved in chemotaxis; for example, the activation of FPR results in the cross-desensitization of CXCR1 and CXCR2 [18]. Thus, it would seem likely that bacterial formyl peptides would mediate neutrophil migration into the mucosa and lumen via the FPR1 even if another chemoattractant first recruited the cells out of the blood. However, testing this hypothesis in mice must take into consideration some of the differences between the mouse versus human family of receptors. In mice, the FPR gene family has at least eight members, with FPR1 the mouse ortholog of human FPR1, although the mouse receptor is approximately 100-fold less sensitive to fMLF than the human receptor [4]. However, the mouse receptor still binds formyl peptides, such as fMIFL [19], and may, therefore, mediate migration to formyl peptides derived from resident bacteria during colitis. We sought to address this issue using FPR1 gene knockout mice.

Following their derivation, the FPR1 gene knockout mice were shown to have increased susceptibility to *Listeria monocytogenes* infection [12], although it was not determined whether this was directly due to a failure to respond to bacterial products or an indirect effect, such as the loss of a host-derived factor that activated leukocytes through FPR1. It was subsequently confirmed that the mouse receptor does directly respond to *Listeria* peptides [19], thereby confirming that the receptor is involved in the host reaction to a pathogen. Additionally, mitochondria-derived* N*-formylated peptides are also potent agonists for FPR1 [20]. In this case and possibly in the mouse also, mitochondrial formylated peptides may activate FPR1 on neutrophils during host cell damage. All of these results suggest that this receptor plays a role in promoting inflammation. Using the FPR1^−/−^ strain, we speculated that formyl peptides generated by bacteria or injured epithelial cells would attract neutrophils into the mucosa and lumen, therefore also contributing to inflammation. However, our data using the FPR1^−/−^ strain suggests that this not the case. While the colonic pathology in acutely inflamed FPR1^−/−^ mice was less than that in C57BL/6 mice, there was a similar number of infiltrating neutrophils in the two strains. One explanation may be the relatively late time at which our measurements were made after discontinuing the DSS treatment. It is possible that the kinetics of infiltrating neutrophils differed, for example, at the time of maximal weight loss between days 7 and 8. Yet both strains still had reduced body weights and inflamed colons at the time the acute experiment was ended, indicating that the mice were ill. Another consideration is a possible indirect effect of formyl peptides acting through FPR1, such as on parenchymal cells, to stimulate other chemoattractants, such as leukotrienes [21]. However, given the similar number of neutrophils that infiltrated the colon in FPR1^−/−^ and C57BL/6 mice and the greater number of neutrophils following two cycles of DSS, there would seem to be no reduction in the chemotactic signals due to the loss of the receptor. We confirmed the latter hypothesis by measuring MIP-2 levels, as a representative CXC chemokine, secreted from the inflamed colons. This family of chemotactic molecules plays a significant role in DSS colitis, and any reduction in levels would likely be associated with reduced inflammation [9, 10]. DSS does lead to a neutrophilia and it is possible that the FPR1^−/−^ mice respond by mobilizing greater numbers of cells from their bone marrow, but we did not evaluate this in the two strains. We did assess whether neutrophils from inflamed FPR1^−/−^mice migrate more effectively to chemoattractants than wildtype cells. Our results demonstrate this is not the case as FPR1-deficient neutrophils migrated to two chemoattractants equally as effectively as neutrophils from C57BL/6 mice. The sum of these findings leads us to conclude that the FPR1 does not mediate neutrophil recruitment, either directly or indirectly, into the DSS inflamed colon.

The data lead us not only to this conclusion, but also to the conclusion that the lack of FPR1 offers some protection from DSS colitis in the acute setting independent of neutrophils. FPR1^−/−^ mice experienced less severe pathological changes than C57BL/6 mice despite both strains having a similar number of infiltrating neutrophils. This result could be explained by the neutrophils becoming activated through the FPR1 and thus contributing to tissue damage in the C57BL/6 mice; however, there are precedents showing that acute DSS colitis occurs in neutrophil-depleted (anti-Gr1 treated) mice [22] and in other models of induced colitis [23]. If DSS colitis is neutrophil independent, then the FPR1 must have roles in colon pathology independent of neutrophils.

The C57BL/6 strain is relatively susceptible to DSS and reportedly continues into a chronic inflammatory condition following a single DSS cycle despite the mice recovering from their weight loss [24]. The C57BL/6 mice in our study recovered their body weight and the signs of chronic inflammation were not apparent even after exposure to a second DSS cycle. Most of the ulcers in these mice had renewed surface epithelium by the end of the study. In contrast, the FPR1^−/−^mice failed to recover their body weight to the same extent as the C57BL/6 mice, suggesting that they failed to fully recover from the first episode of DSS. With the heightened neutrophil infiltrate, it is possible that these cells contribute to the chronic injury despite not contributing to the acute injury or the first DSS cycle. To the best of our knowledge there is no published report on whether neutrophils contribute to the pathology of a second exposure to DSS.

Examination of the FPR1^−/−^ mice colons revealed that one obvious difference from those of the wildtype mice was that the ulcers of the former were not entirely re-epithelialized. We initially thought this was due to a failure of restitution and started to examine annexin A1, a potent mediator of restitution [16]. Annexin A1 levels increase during DSS colitis, with the molecule acting through the FPRL1 [16]; however, we speculated that mucosal levels of annexin A1 might be upregulated by signals through the FPR1 acting as a sensor for invasive bacteria. Furthermore, FPR1 has recently been reported to be present on intestinal epithelium [25, 26] and to play a role in model epithelial cell restitution [27], so the possibility exists that epithelial annexin A1 could be upregulated through FPR1 in mice. Our immunohistochemical detection of annexin A1 proved otherwise, and there was no apparent difference between strains. We complemented the immunohistochemistry with Western blotting of inflamed colonic mucosal scrapings to provide more quantitative data and again the levels between the two strains were similar. It is noteworthy that we detected low levels of annexin A1 in the mouse colon after the second DSS cycle in both mouse strains. These results mean that the lack of FPR1 is not responsible for the apparent failure to replenish epithelial levels of annexin A1 following the second DSS cycle. The almost complete re-epithelialization of the C57BL/6 colons suggests there are sufficient levels of endogenous factors for restitution, including restitution in the presence of neutrophils and after two DSS cycles.

Finally, in the absence of any obvious effect on annexin levels, another explanation for the chronic epithelial damage in FPR1^−/−^mice may be related to an anti-inflammatory role described for fMLF in which fMLF acts directly on intestinal epithelial cells. fMLF, but not other formylpeptides, has been found to stimulate HSP27 in a human colonocyte line, and HSP27 in turn blocks changes in transepithelial electrical resistance and NF-κB activation stimulated by other exogenous mediators [28]. These phenomena were shown to be dependent on the peptide transporter, PepT1, and not on any formyl peptide receptor, although a role for the FPR1 was not ruled out. Thus, HSP27 stimulated by fMLF provides cytoprotective mechanisms. Should the FPR1 be related to this action of a formlypeptide in the mouse, then the cytoprotective effect would be lost in the receptor-deficient mice, possibly explaining the greater epithelial damage in the mice. This mechanism is compatible with a number of bacterial sensing receptors directly providing intestinal epithelial protective processes. Until experiments are planned to pursue this outcome in the FPR1^−/−^ mice, the data here answer the question of whether the FPR1 mediates neutrophil infiltration of the colonic mucosa, and FPR1 is dispensable for this migration.
